# Trend analysis of malaria in urban settings in Ethiopia from 2014 to 2019

**DOI:** 10.1186/s12936-023-04656-6

**Published:** 2023-08-14

**Authors:** Hiwot Teka, Lemu Golassa, Girmay Medhin, Meshesha Balkew, Chalachew Sisay, Endalamaw Gadisa, Dawn M. Nekorchuk, Michael C. Wimberly, Fitsum Girma Tadesse

**Affiliations:** 1 Aklilu Lemma Institute of Pathobiology, Addis Ababa University, Addis Ababa, Ethiopia; 2 Armauer Hansen Research Institute, Addis Ababa, Ethiopia; 3 Abt Associate PMI VectorLink Ethiopia Project, Addis Ababa, Ethiopia; 4 Ethiopian Public Health Institute, Addis Ababa, Ethiopia; 5 Department of Geography and Environmental Sustainability, University of Oklahoma, Norman, USA

**Keywords:** Malaria, Urban, Ethiopia

## Abstract

**Background:**

Urbanization generally improves health outcomes of residents and is one of the potential factors that might contribute to reducing malaria transmission. However, the expansion of *Anopheles stephensi*, an urban malaria vector, poses a threat for malaria control and elimination efforts in Africa. In this paper, malaria trends in urban settings in Ethiopia from 2014 to 2019 are reported with a focus on towns and cities where *An. stephensi* surveys were conducted.

**Methods:**

A retrospective study was conducted to determine malaria trends in urban districts using passive surveillance data collected at health facilities from 2014 to 2019. Data from 25 towns surveyed for *An. stephensi* were used in malaria trend analysis. Robust linear models were used to identify outliers and impute missing and anomalous data. The seasonal Mann-Kendal test was used to test for monotonic increasing or decreasing trends.

**Results:**

A total of 9,468,970 malaria cases were reported between 2014 and 2019 through the Public Health Emergency Management (PHEM) system. Of these, 1.45 million (15.3%) cases were reported from urban settings. The incidence of malaria declined by 62% between 2014 and 2018. In 2019, the incidence increased to 15 per 1000 population from 11 to 1000 in 2018. Both confirmed (microscopy or RDT) *Plasmodium falciparum* (67%) and *Plasmodium vivax* (28%) were reported with a higher proportion of *P. vivax* infections in urban areas. In 2019, *An. stephensi* was detected in 17 towns where more than 19,804 malaria cases were reported, with most of the cases (56%) being *P. falciparum*. Trend analysis revealed that malaria cases increased in five towns in Afar and Somali administrative regions, decreased in nine towns, and had no obvious trend in the remaining three towns.

**Conclusion:**

The contribution of malaria in urban settings is not negligible in Ethiopia. With the rapid expansion of *An. stephensi* in the country, the receptivity is likely to be higher for malaria. Although the evidence presented in this study does not demonstrate a direct linkage between *An. stephensi* detection and an increase in urban malaria throughout the country, *An. stephensi* might contribute to an increase in malaria unless control measures are implemented as soon as possible. Targeted surveillance and effective response are needed to assess the contribution of this vector to malaria transmission and curb potential outbreaks.

## Background

Over half of the world’s population (57%) lived in cities in 2018 [1]. By 2050, the global urban population is projected to double its current size, and most of the increase will be from Asia and Africa [2]. Ethiopia is one of the least urbanized countries with only 23% of its 105 million population estimated to live in cities and towns in 2022 [3]. The percentage of urban residents is projected to increase to 39% by 2050 in Ethiopia [2].

Although living in urban settings generally improves health outcomes of residents [4, 5], urbanization in most developing countries is increasing through informal settlements with poor housing and sanitation that pose health challenges, including infectious diseases [6]. Malaria in sub-Saharan Africa is typically associated with rural settings [5, 7], although it can also thrive in urban areas where suitable larval sites are created because of urban agriculture, expanding construction sites, poorly developed waste management systems in slums, and the adaptation of vectors to polluted urban environments [8–10].

In Ethiopia, malaria transmission is generally low, unstable, and heterogeneous in space and time. In the 2015 Malaria Indicator Survey (MIS) malaria infection prevalence was 0.6% among the urban residents and 1.2% in rural areas [11]. Understanding the factors that contribute to the reported malaria cases from urban areas is very important for planning and implementing appropriate malaria interventions.

*Anopheles arabiensis* is the major malaria vector, whilst *Anopheles pharoensis*, *Anopheles nili* and *Anopheles funestus* play minor roles in malaria transmission in Ethiopia [12]. The larval habitats of these species are temporary water bodies such as rain pools depressions, hoof prints and pools that form at the edges of streams, rivers and lakes [13]. *Anopheles stephensi*, an Asian malaria vector that is expanding its range in the Horn of Africa, thrives in urban areas and prefers to breed in artificial man-made water storage containers and thus has the potential to alter malaria transmission cycles in urban settings [14, 15].

*Anopheles stephensi* is a highly efficient vector of both *Plasmodium falciparum* and *Plasmodium vivax* in urban settings in Asia [16]. First detected in Djibouti in 2012, *An. stephensi* is expanding its range in Africa rapidly [17, 18]. To date, *An. stephensi* has been reported from Ethiopia, Somalia, Sudan, and most recently in Yemen, Nigeria, and Kenya [19–21]. The invasion and establishment of *An. stephensi* is epidemiologically linked with an unusual rise in malaria burden in Djibouti [22]. However, the impact of invasion and establishment of *An. stephensi* in the wider Horn of African region remains unclear.

Following its first detection in 2016 in eastern Ethiopia, subsequent vector surveillance confirmed the presence of *An. stephensi* in 17 of the 25 towns surveyed until 2019 [23, 24]. Considering the trend observed in Djibouti following the invasion of *An. stephensi* and the plasticity of the vector to adapt to varying environments, it was projected to cause substantial increase in cases in Ethiopia if no additional interventions were implemented [15].

The current study aimed to quantify historical malaria burden in urban settings of Ethiopia and assess the trend using routine Public Health Emergency Management (PHEM) surveillance data between 2014 and 2019 in areas where the presence of *An. stephensi* has been reported.

## Methods

### Study context

In Ethiopia, health service delivery is a three-tier system that includes primary health care units (PHCU) comprising a district hospital, health centre and five satellite health posts at the lowest level. Zonal hospitals are at the second tier with specialized referral hospitals at the top tier [25]. At health centres and hospitals, microscopy is used to diagnose malaria while malaria rapid diagnostic tests (RDTs) are used at the community, health post level [26]. Malaria is among 20 diseases rapidly reportable to the Public Health Emergency Management (PHEM) Center at the Ethiopian Public Health Institute (EPHI). The centre is tasked to coordinate efforts to improve the preparedness of the health sector to prevent and/or reduce the public health consequences of emergencies. Malaria cases are reported weekly from each health facility and then are collated at district, zonal and regional level and sent to PHEM. Then, the analysed information at all levels will be used to inform preparedness, prevention, and response to consequences of epidemic threats [27].

### Data collection and management

Electronic records of national malaria surveillance data reported from 2014 to 2019 were obtained from the EPHI to explore the burden of malaria in urban settings in Ethiopia. Counts of total malaria cases (confirmed plus clinically diagnosed), and confirmed positive cases for *P. falciparum*, and *P. vivax* were included in the report (mixed infections are not reported separately). Eight hundred thirty-four districts reported through the PHEM system and 150 of these are urban districts. The districts were not categorized as rural or urban in the PHEM dataset. A rural-urban category was created using administrative classification data obtained from regional health bureaus. The Ethiopian government classifies areas as urban districts if at least one of the following criteria is met: localities with at least 2000 inhabitants, localities with at least 1000 inhabitants that primarily depend on non-agriculture activities, all administrative capitals (regional, zonal, or district), and localities with an Urban Dweller’s Association [28]. District level aggregated data was used to assess the burden of malaria in the urban districts. In addition, 25 towns were selected for time series trend analysis based on reported presence (17 towns) or absence (8 towns) of *An. stephensi* between 2014 and 2019. Time series graphs of annual parasite incidence (API) were generated to visualize annual malaria trends at the national level, stratified by urban and rural districts. For the 25 towns surveyed for *An. stephensi*, monthly time series of anomaly-removed malaria case numbers (total malaria, *P. falciparum*, and *P. vivax*, separately) were generated and then depending on the analysis, aggregated to the urban/rural classification. Seasonal Mann-Kendall tests based on monthly malaria case summaries were used to test for monotonic increases or decreases in malaria cases. Population data were projected from 2007 census data based on the estimated national growth rate and used to calculate annual parasite incidence (API) per 1000 population [28].

In addition, for each of the 17 towns with reported presence of *An. stephensi*, descriptive climate data, including average yearly mean land surface temperature (LST), and average yearly total precipitation, was summarized from Moderate Resolution Imaging Spectroradiometer (MODIS) data and Global Precipitation Mission (GPM) data using the REACH Google Earth Engine script application [29] to provide environmental context of these areas.

### Imputation of missing and anomalous data

In the PHEM dataset, reported weekly malaria case data are sometimes missing or unexpectedly low. For example, zero cases may be reported in a district that typically has hundreds of cases per week, and it is reasonable to suspect the count should have been reported as missing data instead of zero. Thus, robust linear models were used to identify these outliers and estimate the expected number of cases in weeks with missing or anomalous data for every district in Ethiopia. The models were fitted using the rlm function with default options from the MASS package in R [29]. The following model was fitted to the malaria data from each district:

$${\text{log}}\left( {{\text{c}}_{{{\text{y}},{\text{w}}}} } \right)\, = \,{\text{b}}_{0} \, + \,{\text{s}}\left( {\text{y}} \right)\, + \,{\text{s}}\left( {\text{w}} \right)$$

where c is the total number of malaria cases for each year (y) and week (w) combination, b_0_ is an intercept term, s(y) is a smoothed multi-year trend based on a cubic spline, and s(w) is a seasonal trend based on a cyclical cubic spline [30]. Separate models were fitted for total malaria cases, confirmed *P. falciparum* cases, and confirmed *P. vivax* cases. For weekly records with missing malaria case counts, the missing values were replaced by fitted values from the regression model. Reported weekly case numbers that were more than three standard errors below the fitted value in a given week were considered anomalous and were replaced by fitted values from the regression model. All other raw data values were retained in the final dataset.

The percent of weeks with one or more missing or anomalous data values in the overall dataset declined over time from 16.7% to 2014 to 9.8% in 2019. Kebridehar, one of the 17 towns where *An. stephensi* was detected, had missing data for most of the years (2017, 2018 and 2019). Overall, there were no data reported in 8.2% of all weeks, and anomalous data values were detected in 4.2% of the weeks for which data were reported. The majority (82.2%) of these anomalous data values were zeroes, which in most cases appeared to result from incorrect labelling of missing data.

### Data analysis

Descriptive data analysis was performed using GraphPad PRISM 6.0 to determine the annual incidence of malaria. The anomaly-removed weekly data was aggregated to monthly data to conduct a seasonal Mann-Kendall test for monotonic increase or decrease trends in the total number of malaria cases, total number of *P. falciparum*, and *P. vivax*. This approach to account for the strong seasonality of malaria cases and to increase statistical power compared to the standard Mann-Kendall test [31]. An alpha-level of 0.05 was used to determine statistical significance. The Mann-Kendall trend tests were carried out in R software using the trend package [32]. A significant trend provided evidence of a consistent increase or decrease in malaria cases over the 6-year study period. Towns were labelled with strata based on the annual parasite incidence (API) per 1000 population, according to the national strategic plan [26]. These stratification categories include high endemic (API ≥ 50), medium (API ≥ 10 < 50), low (API > 5 < 10), very low (API > 0 ≤ 5), and malaria-free areas (API = 0).

## Results

### Malaria burden in urban settings in Ethiopia

During the period from 2014 to 2019, there were 9,468,970 total cases reported through the PHEM system. Among these, 6.34 million (67%) were *P. falciparum* cases, 2.63 million (28%) were *P. vivax* cases, and the remaining 492,666 (5%) were clinical cases treated without confirmation. Of the total cases during these periods, 1.45 million cases (15.3%) were reported from urban districts. Both species of *Plasmodium* parasites were reported from urban settings, with a higher proportion of *P. vivax* infection (33%, 470,564 of 1.45 million) compared to its nationwide contribution.

**Fig. 1:**
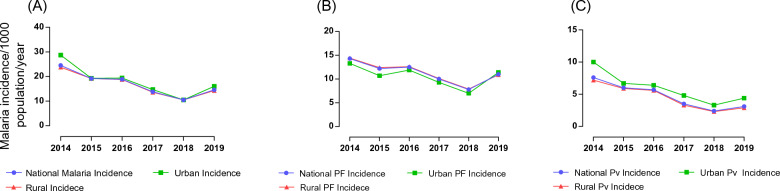
National, urban, and rural incidence of **A** total malaria, **B** total *P. falciparum*, **C** total *P. vivax* malaria in Ethiopia

The overall national trend in total malaria declined by 53% from 2014 to 2018; incidence dropped from 25 cases per 1000 population in 2014 to 10 cases per 1000 population in 2018. There was a slight increase in malaria incidence in 2019 to 15 cases per 1000 population. The malaria cases reported from urban settings followed similar trends (Fig. 1A). The incidence in urban areas was 29 cases per 1000 population in 2014, 11 cases per 1000 in 2018, and 16 cases per 1000 in 2019. The incidence of *P. falciparum* was higher in rural than urban districts from 2014 to 2018 (Fig. 1B). On the other hand, the incidence of *P. vivax* cases was consistently higher in urban settings than rural areas (Fig. 1C).

The seasonal Mann-Kendall tests confirmed the overall declining trends of total malaria, *P. falciparum*, and *P. vivax* malaria in Ethiopia from 2014 to 2019 despite the increase observed from 2018 to 2019. These declining trends occurred in both rural and urban districts (Table 1).

**Table 1:** Seasonal Mann-Kendall trend test results for national level summaries

Level	S (tot)	Z (tot)	P (tot)	S (pf)	Z (pf)	P (pf)	S (pv)	Z (pv)	P (pv)
National	−102	−5.477	< 0.001	−76	−4.067	< 0.001	−130	−6.996	< 0.001
Urban	−108	−5.803	< 0.001	−76	−4.067	< 0.001	−134	−7.213	< 0.001
Rural	−98	−5.261	< 0.001	−60	−3.200	< 0.001	−126	−6.779	< 0.001

* S* Seasonal Mann-Kendall statistic, *Z* Z-score of the seasonal Mann-Kendall test, *P* P-value of the seasonal Mann-Kentall test, *tot* total malaria, *pf* *Plasmodium falciparum*, *pv* *Plasmodium vivax*

### Retrospective analysis of historical malaria burden in urban areas with reports of *An. stephensi* invasion

**Fig. 2:**
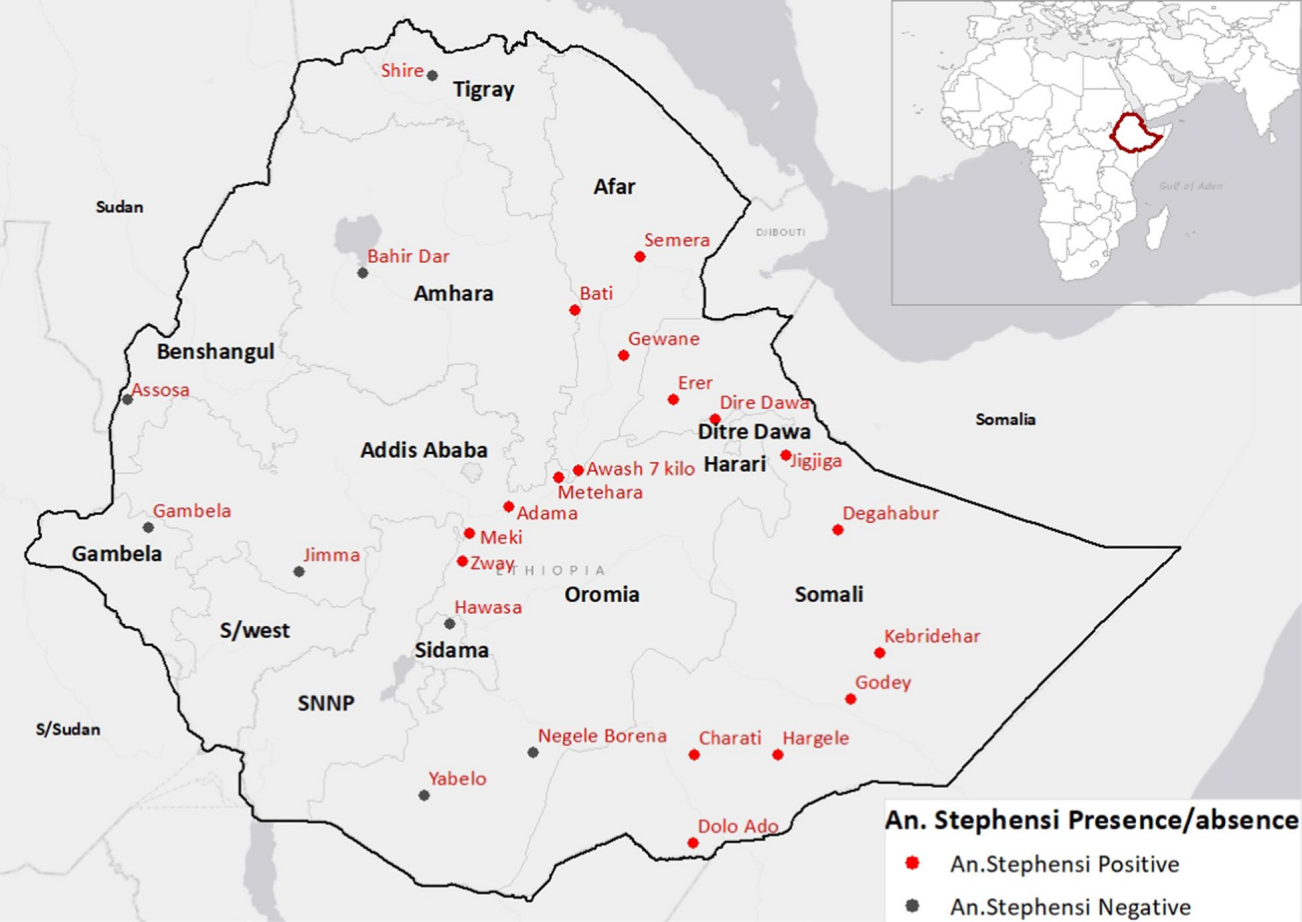
Map of the towns where *An. stephensi* had been surveyed

Of the 25 sites where entomological surveillance was conducted between 2016 and 2019, *An. stephensi* was reported from 17 towns in central and eastern Ethiopia [23, 24]. These towns are from five administrative regions (Fig. 2; Table 1). The towns are located along the main route to and from Djibouti and their altitude ranges from 260 to 1643 meters above sea level (masl). Most towns are located in the arid areas of Somali and Afar regions with annual average precipitation of 313–943 mm and average yearly temperature ranging between 23 and 35 ^o^C. The population size in these towns ranges from 18,160 to 468,282 [3]. Most of the towns (n = 15) are trade and transportation hubs. Among the 17 towns, Adama, Meki and Ziway are close to the cargo (dry) port at Modjo.

In 2019, a total of 19,804 malaria cases were reported in these 17 towns, among which 10,998 (56%) were *P. falciparum* and 3,794 (19%) were *P. vivax* cases, and the remaining were clinically treated as malaria (25%, 5,012). The towns were labelled with their identified transmission strata (Table 2). Most of the towns (n = 11) were in the medium transmission stratum except four very low (Ziway, Adama, Dire Dawa and Jigjiga), one low (Godey), and one high transmission town (Metehara).

**Table 2:** Description of study areas

Region	Town	Annual average rainfall	Annual mean temperature (C°)	Altitude masl	Area (Km2)*	Population in 2019	API per 1000 in 2019	Transmission category	Year *An. stephensi* reported
Afar	Awash Sebat Kilo	818.4	31.2	986	16	18,160	41.4	Medium	2018
	Gewane	659.3	33.8	618	4	38,785	12.6	Medium	2018
	Semera	358	35.1	432	26	41,626	40.4	Medium	2018
Amhara	Bati	942.9	23.4	1502	3.4	37,557	18.9	Medium	2018
Dire Dawa	Dire Dawa	932.6	28.5	1276	35.4	468,282	1.9	Very low	2018
Oromia	Meki	922.6	24.2	1636	8.4	207,349	11.3	Medium	2019
	Metehara	847.5	25.5	947	2.5	32,536	52.8	High	2019
	Ziway	925.4	24.7	1643	9.3	146,521	1.7	Very low	2019
	Adama	937.4	25.1	1610	74.7	368,694	2.5	Very low	2019
Somali	Degehabur	525.3	29.1	1044	7.7	42,036	11.3	Medium	2018
	Erer	705.7	33.1	1107	2	69,129	27.7	Medium	2018
	Godey	313.1	32.9	260	16.8	60,049	9	Low	2018
	Jigjiga	883.8	25.4	1652	19.7	164,054	3.5	Very low	2018
	Kebridehar	330.8	30.7	1609	19.8	40,964	11.4	Medium	2016
	Chereti	421.1	32.4	290	2.4	128,409	10.4	Medium	2021
	Dollo Ado	380.7	32.0	538	24.8**	149,795	13.7	Medium	2021
	Hargele	336.7	31.5	502	4**	110,799	21.7	Medium	2021

*The area of the towns are measured using delineations from google maps

** The last 2 towns are measured using a polygon overlaid on the town using google earth polygon measure tool

Seven of the towns, Meki, Metehara, Erer Gota, Semera, Haregele, Dollo Ado and Chereti reported more than 1,000 cases per year (ranges from 1,338 to 2,405). The remaining 10 towns: Adama, Dire Dawa, Jigjiga, Bati, Awash Sebat Kilo, Gewane, Kebridar, Degehabour, Godey and Ziway reported fewer than 1,000 cases, ranging from 253 to 931.

**Fig. 3:**
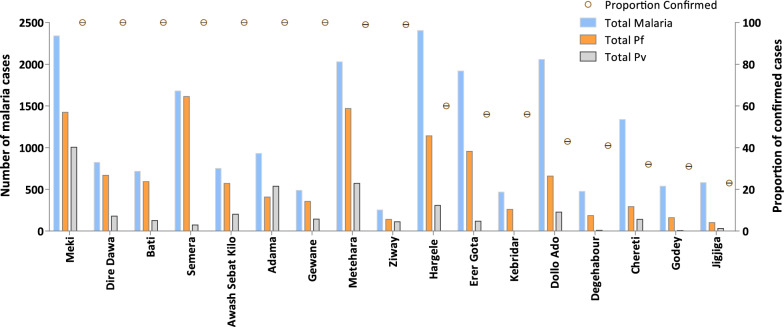
Number of malaria cases and proportion of confirmed cases reported in 2019 in 17 towns where *An. stephensi* was detected

All towns outside of the Somali region (n = 9) performed confirmatory tests on the majority of suspected cases (> 99% confirmatory tests). The eight towns in Somali region (Erer Gota, Jijiga, Degehabour, Godey, Haregele, Dollo Ado, Chereti and Kebridar) conducted confirmatory tests only on 60% of suspected cases or less (range 23–60%) (Fig. 3).

**Fig. 4:**
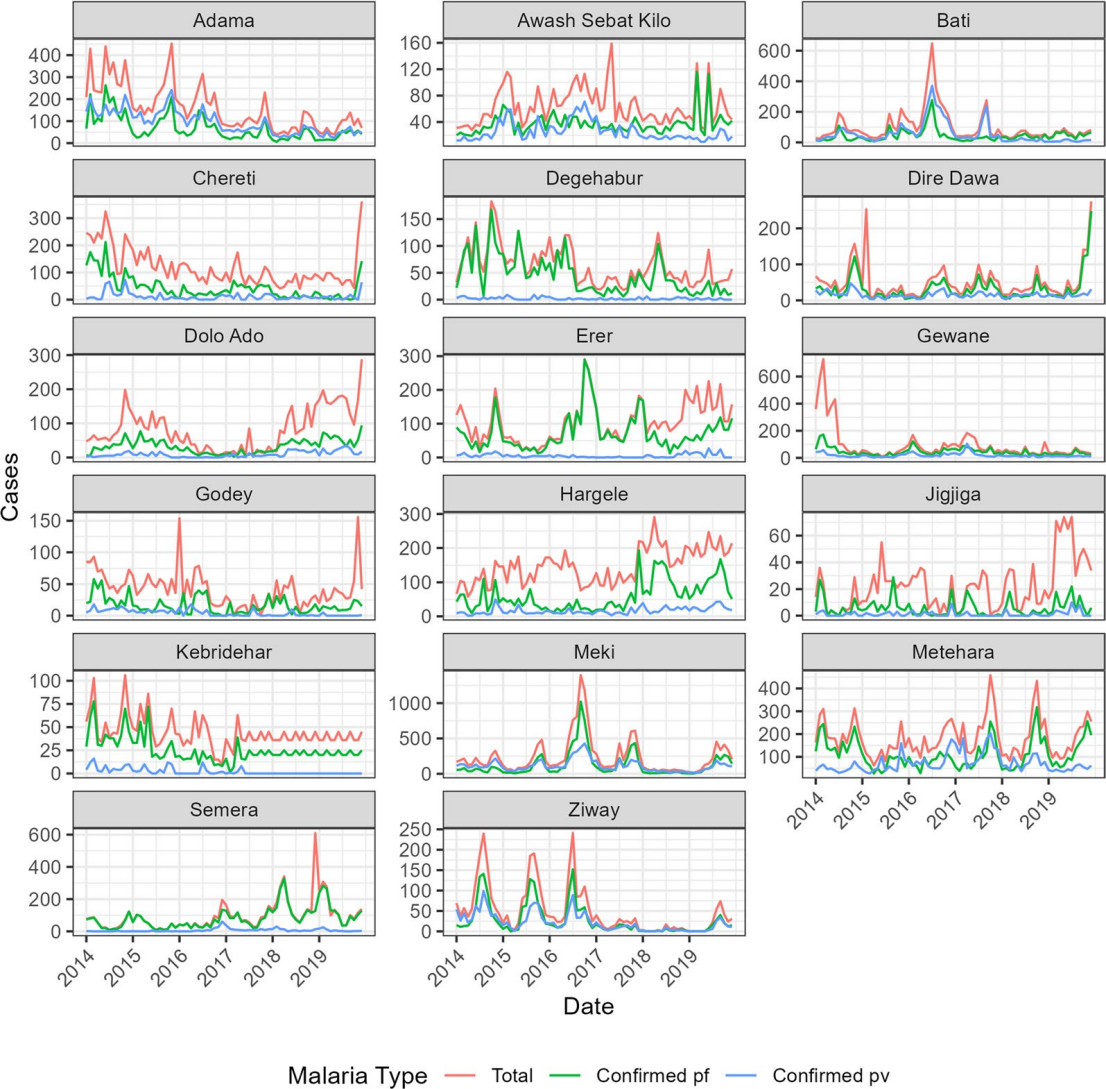
Monthly malaria time series for towns where *An. stephensi* has been detected

The seasonal Mann-Kendall tests confirmed the trends that were observed in the time series graphs. Declining trends were found in many of the towns where *An. stephensi* is known to be present (Table 3; Fig. 6). Adama, Bati, Chereti, Degehabur, Gewane, Godey, Kebridehar, Meki, and Ziway all had declining trends for at least one of the three malaria variables. In contrast, Dolo Ado, Erer Gote, Hargele, Jigiiga, and Semera had increasing trends. Erer, Hargele, Jigiiga, and Semera exhibited positive trends for total malaria cases, whereas Dolo Ado, Hargele, and Semera had positive trends for confirmed *P. falciparum* and *P. vivax*. Kebridehar had missing data for most of the years 2017, 2018 and 2019. The values shown in the graph for these years were all imputed using the robust regression models (Fig. 4; Table 3).

**Table 3:** Seasonal Mann-Kendall trend test for towns with *An. stephensi* present

Town	S (tot)	Z (tot)	P (tot)	S (pf)	Z (pf)	P (pf)	S (pv)	Z (pv)	P (pv)
Adama	−138	−7.43	< 0.001	−131	−7.061	< 0.001	−138	−7.43	< 0.001
Awash Sebat Kilo	−10	−0.488	0.625	8	0.382	0.703	−34	−1.806	0.071
Bati	−28	−1.469	0.142	35	1.852	0.064	−83	−4.454	< 0.001
Chereti	−98	−5.276	< 0.001	−99	−5.323	< 0.001	−13	−0.66	0.509
Degehabur	−76	−4.079	< 0.001	−108	−5.803	< 0.001	−43	−2.479	0.013
Dire Dawa	−16	−0.822	0.411	5	0.219	0.827	−20	−1.046	0.296
Dolo Ado	37	1.955	0.051	38	2.013	0.044	63	3.387	0.001
Erer	72	3.851	< 0.001	37	1.955	0.051	−29	−1.561	0.118
Gewane	−42	−2.23	0.026	−34	−1.79	0.074	−24	−1.255	0.210
Godey	−56	−2.992	0.003	−41	−2.173	0.030	−95	−5.336	< 0.001
Hargele	92	4.95	< 0.001	68	3.644	< 0.001	87	4.732	< 0.001
Jigjiga	51	2.716	0.007	−4	−0.167	0.867	2	0.064	0.949
Kebridehar	−50	−2.731	0.006	−40	−2.173	0.030	−95	−5.910	< 0.001
Meki	−39	−2.064	0.039	−17	−0.869	0.385	−62	−3.308	0.001
Metehara	−2	−0.054	0.957	10	0.490	0.624	23	1.198	0.231
Semera	108	5.837	< 0.001	103	5.54	< 0.001	53	2.936	0.003
Ziway	−116	−6.274	< 0.001	−104	−5.659	< 0.001	−129	−6.993	< 0.001

* S* Seasonal Mann-Kendall statistic, *Z* Z-score of the seasonal Mann-Kendall test, *P* P-value of the seasonal Mann-Kentall test, *tot* total malaria *pf* *Plasmodium falciparum*, *pv* *Plasmodium vivax*

**Fig. 5:**
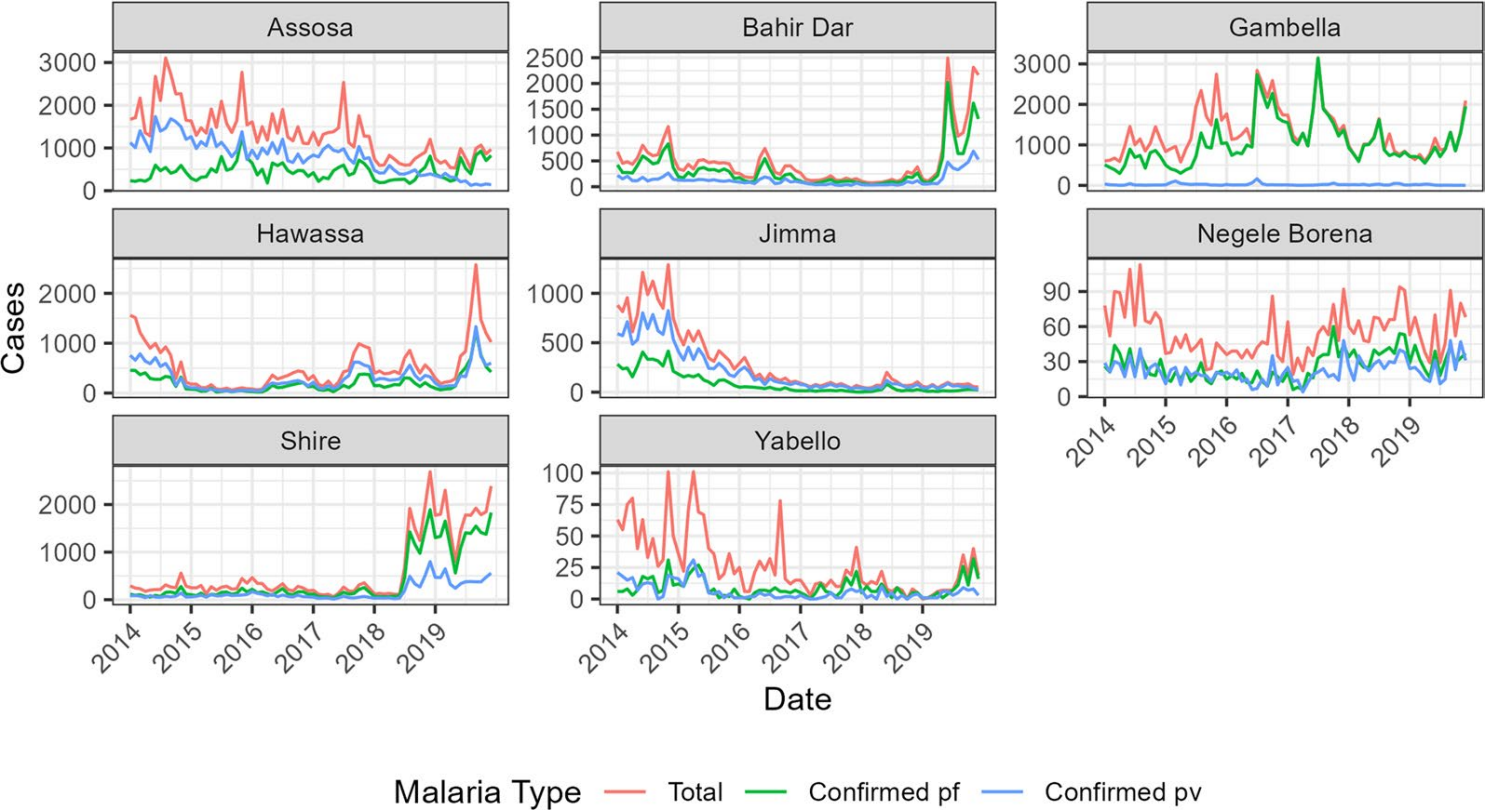
Monthly malaria time series for towns where *An. stephensi* has not been detected

**Fig. 6:**
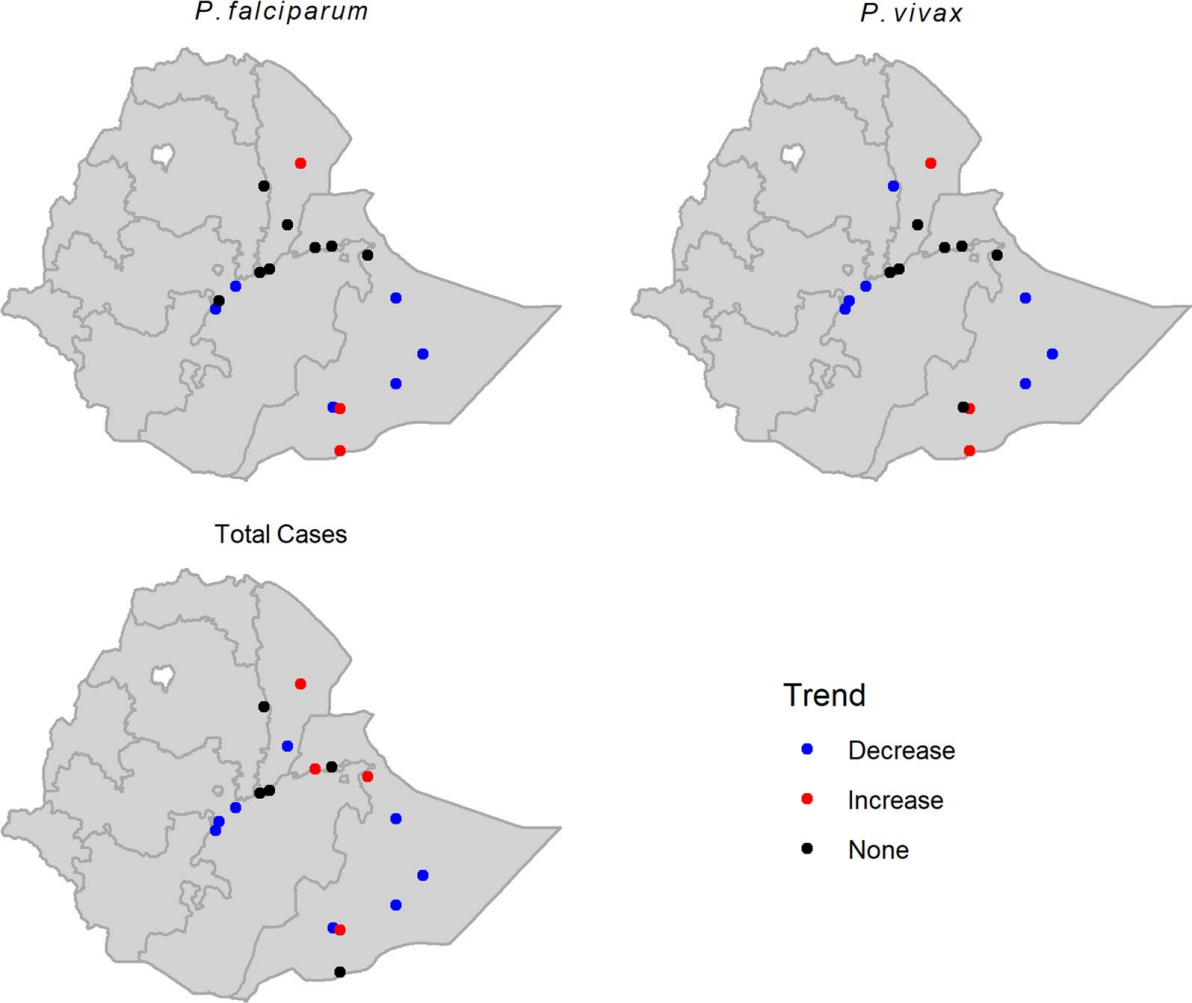
Maps of decreasing and increasing malaria trend in seventeen towns where *An. stephensi* was reported

During the entomological surveillance, there were 8 towns (Assosa, Bahir Dar, Jimma, Yabello, Hawassa, Gambella, Negele Borena and Shire) that were negative for *An. stephensi*. Among these, four towns (Assosa, Bahir Dar, Jimma, and Yabello) showed significant decreasing trends for total malaria cases, two towns (Bahir Dar and Jimma) for total *P. falciparum* malaria cases, and four towns (Assosa, Bahir Dar, Jimma, Yabello) for total *P. vivax* malaria cases (Fig. 5; Table 4). Two of the eight towns (Hawassa and Shire) had significantly increasing trends for total malaria cases and *P. vivax* cases. Four out of the eight towns (Gambella, Hawassa, Negele Borena, and Shire) showed significantly increasing trends for *P. falciparum* cases (Fig. 5; Table 4). Maps of sites with positive, negative, and non-significant malaria trends are summarized in Fig. 6.

**Table 4:** Seasonal Mann-Kendall trend test for towns with *An. stephensi* absent

Town	S (tot)	Z (tot)	P (tot)	S (pf)	Z (pf)	P (pf)	S (pv)	Z (pv)	P (pv)
Assosa	−132	−7.104	< 0.001	−10	−0.488	0.625	−148	−7.972	< 0.001
Bahir Dar	−63	−3.367	0.001	−63	−3.367	0.001	−54	−2.874	0.004
Gambella	−12	−0.597	0.551	43	2.281	0.023	−19	−0.981	0.327
Hawassa	48	2.549	0.011	44	2.332	0.020	48	2.549	0.011
Jimma	−140	−7.538	< 0.001	−130	−7.017	< 0.001	−142	−7.647	< 0.001
Negele Borena	−8	−0.380	0.704	40	2.121	0.034	8	0.382	0.703
Shire	44	2.332	0.020	65	3.476	0.001	42	2.224	0.026
Yabello	−114	−6.146	< 0.001	−22	−1.164	0.244	−47	−2.573	0.010

* S* Seasonal Mann-Kendall statistic, *Z* Z-score of the seasonal Mann-Kendall test, *P* P-value of the seasonal Mann-Kentall test, *tot* total malaria, *pf* *Plasmodium falciparum*, *pv* *Plasmodium vivax*

## Discussion

Following the detection of *An. stephensi* in Ethiopia in 2016 and its subsequent establishment along the major transportation corridor, this study examined for the first time the trend of urban malaria in Ethiopia in towns and cities with and without confirmed presence of *An. stephensi*. The overall national malaria incidence showed a declining trend between 2014 and 2018 but had a slight increase in 2019. Urban areas contributed 15% of the reported malaria cases and had similar declining trends as in non-urban areas during the study period. However, no clear difference was observed in case incidence over the years between cities/towns with and without *An. stephensi*. It is important to take note that all reported cases in urban settings might not necessarily result from local urban transmission. Some might be imported cases from the neighbouring rural-peri-urban areas, or they could be travel related [33, 34].

The overall decline in malaria cases may be attributed to the widespread implementation of prevention and control interventions in the country [35]. The rise in incidence in 2019 could be linked with the internal displacement of communities due to civil unrest and natural disasters such as flooding [36]. Screening of displaced populations in camps have shown increased prevalence of malaria [37]. Additionally, the development of *P. falciparum* histidine rich protein (*pfhrp2/3*) deletion may increase the parasite pool in population through escaping diagnostic tool and thus contribute to the increase in cases [38].

The proportion of *P. vivax* cases was found to be higher in urban settings (33%) compared to the national proportion (28%). This could be due to the unique biology of *P. vivax* that allows it to remain quiescent in the liver for months or years and then reemerge, which allows the parasite to persist. Urban dwellers, once infected with *P. vivax*, may repeatedly appear in the health facilities with unpredictable relapsing episodes that make them challenging to the control and elimination measures. In the background of low local mosquito driven transmission, the contribution of *P. vivax* to the overall infection burden might remain high. Additionally, *P. vivax* parasites can complete their life cycle within the mosquitoes at lower temperatures compared to *P. falciparum* and is detected across wide geographic ranges. Towns and cities were often established at higher altitude settings where most of the population settles [39].

The findings in this study show that confirmation of malaria (by microscopy or RDTs) was performed for more than 99% of the cases in nine towns. These could be due to the high availability and accessibility of malaria diagnostics and treatment services in urban areas [40] or due to underreporting of all suspected malaria cases in the PHEM system thus skewing the denominator [41]. However, eight towns, all from Somali Region, reported confirmation in less than 60% of malaria cases. This might be related to the limited availability of diagnostic services for malaria in the Somali region [40].

Trend analysis for the towns with *An. stephensi* have shown no increases of reported malaria cases in 13 towns except Erer Gote, Hargele, Jijiga, Dolo Ado and Semera. Four towns where *An. stephensi* was not found (i.e. Gambella, Hawassa, Negele Borena, and Shire) also had significant trends of increases in malaria cases. Although the introduction of *An. stephensi* may have contributed to some of the local increases, these findings suggest that other drivers are also playing important roles. These factors may have included increased utilization of health services, improved reporting, increases in transmission due to environmental and emerging biological factors, and population movement [34, 42, 43]. The likely explanation for why malaria cases did not increase in towns with *An. stephensi* could be the overall low malaria incidence in the area and low sporozoite infection rate of mosquito vectors in the urban districts. In 2019, entomological monitoring activity showed low sporozoite infection rate with 0.4% for Dire Dawa City and 0.3% for Kebridar town. Additionally, blood meal source analyses conducted in these two urban settings have shown low feeding rates of *An. stephensi* on humans. Only 0.25% of the mosquitoes were found fed on human blood in Dire Dawa and while none were found in Kebridar [44]. Both low parasite and vector indices affect transmission, as there needs to be a critical mass of vector and parasite presence in the area to drive an increase in transmission. Another explanation may be the prevention and control interventions implemented in the area. All of the towns are under 2000 m elevation and are designated as malarious [26]. According to the national strategic plan, all these towns were eligible for long-lasting insecticidal nets (LLINs) and free diagnosis and treatment services [26]. These ongoing interventions may have limited the potential for *An. stephensi* to influence malaria transmission.

The use of surveillance data which was reported at the facilities might underestimate the true burden of urban malaria. The reporting location is where the patient was diagnosed, not necessarily where transmission occurred. Reported urban malaria cases might not all be indigenous, and include some cases that come from the neighbouring rural areas or might be travel related [34]. The use of aggregated data at district level precluded analysis at a granular village “kebele” level and made the assessment of malaria burden in the urban setting challenging as some of the districts may have both urban and rural “kebeles” [24]. Since this information is not captured in the existing PHEM reports, such information might need to be included in future revision of surveillance system. Furthermore, the data do not include most of the cases diagnosed at private health facilities where 25% of febrile patients seek treatment for malaria [11].

Surveillance data are also affected by treatment-seeking behaviour, and testing rate [45] which are not factored into the current analysis. Missing values in the surveillance datasets, including many that were coded as zero values, also presented a challenge. Although the method for data screening and imputation helped to reduce potential biases, the missing and underreported case counts likely reduced the ability to detect trends. Finally, the PHEM data extended only through 2019. Given the relatively short 6-year time series, the statistical analysis is limited to relatively straightforward and robust methods for monotonic trend detection from 2014 to 2019. The influence of *An. stephensi* on malaria transmission may change in future if this invasive mosquito becomes more established and increases in abundance.

## Conclusion

An increase in malaria cases from 2014 to 2019 was documented in 5 towns where *An. stephensi* has been surveyed: Semera, Hargele, Dollo Ado, Erer Gote and Jijiga. The presence of *An. stephensi* may exacerbate the situation, thus, close monitoring of trends in malaria cases are important to identify and respond to outbreaks that may be caused by the newly introduced malaria vector. However, declines in malaria also occurred in some cities with *An. stephensi* and increases occurred in some cities that did not have *An. stephensi*. These conditions are suggestive of other factors that may also be influencing these trends. Early diagnosis, treatment of cases and surveillance will be of a paramount importance to mitigate the impacts that could arise from the newly established malaria-transmitting vector.

## Data Availability

Data supporting the conclusions of this article are provided within the article. Raw data can be made available upon request to the Ethiopian Public Health Institute, owner of the surveillance data.

## Abbreviations

API: Annual parasite incidence
An: Anopheles
EPHI: Ethiopian Public Health Institute
FMOH: Federal Ministry of Health
GPM: Global Precipitation Mission
LST: Land surface temperature
masl: Metres above sea level
MIS: Malaria indicator survey
MODIS: Moderate resolution Imaging Spectroradiometer
PHCU: Primary health care unit
PHEM: Public Health Emergency Management
RDT: Rapid diagnostic test
WHO: World Health Organization
